# PIEZO channels link mechanical forces to uterine contractions in parturition

**DOI:** 10.1126/science.ady3045

**Published:** 2025-11-13

**Authors:** Yunxiao Zhang, Sejal A. Kini, Sassan A. Mishkanian, Oleg Yarishkin, Renhao Luo, Saba Heydari Seradj, Verina H. Leung, Yu Wang, M. Rocío Servín-Vences, William T. Keenan, Utku Sonmez, Manuel Sanchez-Alavez, Yuejia Liu, Xin Jin, Darren J. Lipomi, Li Ye, Michael Petrascheck, Antonina I. Frolova, Sarah K. England, Ardem Patapoutian

**Affiliations:** 1 Department of Neuroscience, Dorris Neuroscience Center, The Scripps Research Institute; San Diego, CA, 92037, United States; 2 Howard Hughes Medical Institute; Chevy Chase, MD, 20815, United States; 3 Aiiso Yufeng Li Family Department of Chemical and Nano Engineering, University of California San Diego; La Jolla, CA, 92093, United States; 4 Department of Chemical Physiology, The Scripps Research Institute; La Jolla, CA, 92037, United States; 5 San Diego Biomedical Research Institute, San Diego, CA, 92121, United States; 6 Universidad Autónoma de Baja California, Facultad de Medicina y Psicología, Mesa de Otay, 22424, Tijuana, Mexico; 7 WashU Medicine, Department of Obstetrics and Gynecology, Center for Reproductive Health Sciences; Saint Louis, MO, 63110, United States

## Abstract

Mechanical forces are extensively involved in pregnancy and parturition, but their precise roles and mechanisms remain poorly understood. We identified mechanically activated ion channels PIEZO1 and PIEZO2 as key mechanotransducers required for labor progression. Genetic deletion of *Piezo1* and *Piezo2* in mice resulted in weakened uterine contractions and severe parturition defects. Tissue-specific knockouts revealed that deletion in either the uterus or sensory neurons alone caused modest defects, whereas combined loss markedly impaired labor, demonstrating additive effects. Single-nuclei sequencing indicated that loss of PIEZO function reduced expression of connexin43 (*Gja1*), a gap junction protein in uterine smooth muscle cells, suggesting a mechanistic link to impaired contraction. These findings highlight the critical role of PIEZO channels in mechanotransduction during parturition and suggest therapeutic targets for labor dysfunction.

Mechanotransduction, the cellular response to mechanical stimuli, is essential for a wide range of biological processes, from tactile perception to the maintenance of tissue homeostasis (1–4). Although both mechanical cues and chemical ligands influence physiological functions across multiple organ systems, including cardiovascular (5), respiratory (6) and gastrointestinal activities (7), the extent to which mechanical forces contribute under different physiological conditions remains less well characterized.

Pregnancy and parturition are key physiological events in which mechanical forces play a critical role alongside hormonal and other biochemical signals. During pregnancy, the human uterus can increase its volume capacity by up to 500 times (8), and parturition requires coordinated uterine contractions and extensive cervical remodeling—processes inherently accompanied by substantial changes in mechanical forces (9). Although hormones, particularly progesterone, are well-documented regulators of pregnancy (10, 11), evidence indicates that mechanical cues also play a critical and complementary role. As early as 1941, studies by Ferguson et al. suggested that mechanical distention at the vagina stimulates uterine contractions as parturition approaches (12). Since then, accumulating evidence has linked mechanical cues to uterine hypertrophy, myometrial contractility, and cervical softening (9, 13), all of which are essential for successful reproduction in mammals.

Multiple signaling mechanisms —including mitogen-activated protein kinase (MAPK) signaling (14, 15), Hippo/YAP signaling (16), matrix metalloproteinases (MMPs) (17), and ion channels (18–20)—have been implicated in mechanosensation. However, since most of these pathways are also activated by biochemical cues, it remains challenging to determine whether the effects of disrupting these pathways are solely due to the loss of mechanotransduction. In contrast, mechanically-activated ion channels PIEZO1 and PIEZO2 are directly gated by forces, and unlike many other signaling pathways, lack known endogenous chemical ligands that modulate their activity (21). Thus, phenotypes associated with PIEZO loss-of-function likely result specifically from disrupted mechanical force sensing. We demonstrate that PIEZO1 and PIEZO2 are specifically required for effective uterine contractions and successful parturition. Knockout of these channels resulted in impaired uterine contractions and prolonged labor, suggesting mechanotransduction as a critical regulatory mechanism for parturition.

## Results

### Distinct expression patterns of *Piezo1* and *Piezo2* in the reproductive system

**Figure 1.**
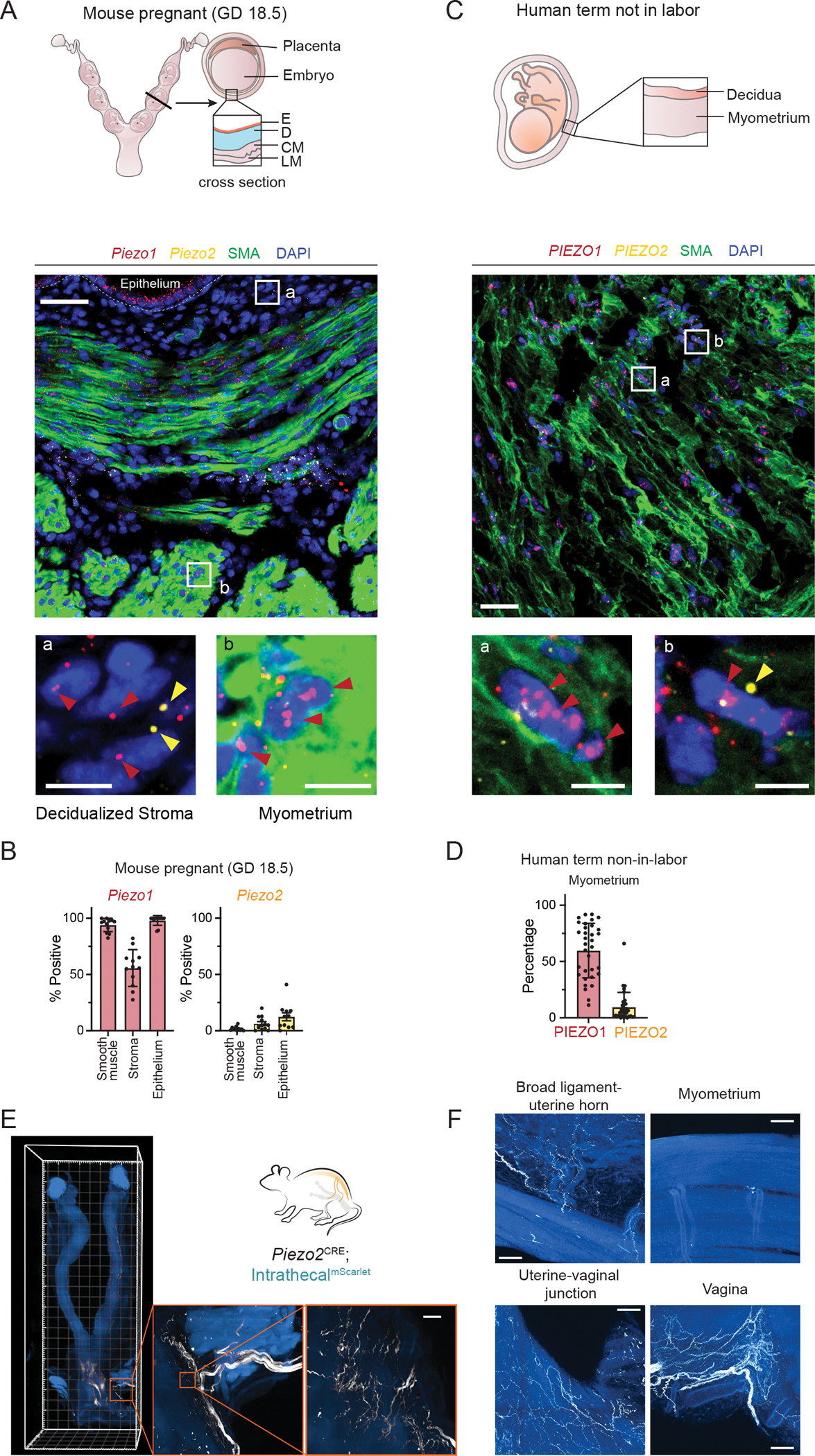
PIEZO1 and PIEZO2 expression in the uterus. (A) Expression of *Piezo1* and *Piezo2* transcripts in mouse uterus at late pregnancy (gestational day 18.5). Scale bar: 50 μm in the overview, 10 μm in the zoomed inset. The percentage of positive cells is quantified in (B). Representative images from 3 animals. Cells quantified from 12 images. E: epithelium. D: Decidualized stroma. CM: circular myometrium. LM: longitudinal myometrium. (C) Expression of *PIEZO1* and *PIEZO2* transcripts in human term-not-in-labor. Scale bar: 50 μm in the overview, 10 μm in the zoomed inset. The percentage of positive cells is quantified in D. Representative images from 3 donor specimens. Cells quantified from 33 images. (E) *Piezo2*-positive sensory innervation was labeled by intrathecal delivery of MacPNS1 AAV virus encoding CAG-DIO-mScarlet into *Piezo2*^cre^ animals. The *Piezo2* sensory fibers were observed primarily in the lower reproductive tract. (F) Confocal imaging revealed Piezo2-positive fibers in the vaginal walls and at the junction between uterine body and vagina but rarely within the uterine horns. *Piezo2* fibers were also present in the broad ligament and around major vasculature towards the horns. Images were collected from 3 animals. Scale bar: 100 μm.

We first characterized the distribution of *Piezo1* and *Piezo2* in the mouse reproductive system using single-molecule fluorescence in situ hybridization (smFISH). At gestational day 18.5 (GD18.5, ~1 day before parturition), *Piezo1* mRNA was widely detected across most cell types within the uterine horn, whereas *Piezo2* expression was confined to a small subset of cells within the decidualized stroma (Fig. 1, A and B). A similar expression pattern was observed in non-pregnant uterine horns at estrus (Fig. S1, A and B), a finding corroborated by published single cell sequencing data (22). Moreover, the overall expression patterns of both *Piezo1* and *Piezo2* remain largely unchanged throughout different stages of the estrous cycle (Fig. S1 C and D). Notably, among the cell types analyzed, only the lymphatic endothelial cells (LECs), which are primarily localized between circular and longitudinal myometrium at late pregnancy (GD18.5), showed high expression of *Piezo2* (Fig. S1E). In the cervix, while *Piezo1* was broadly expressed across various cell types, *Piezo2* expression was predominantly observed in epithelial cells (Fig. S1, F to H).

We next assessed *PIEZO1* and *PIEZO2* expression in human reproductive tissues. In non-labor term myometrium, *PIEZO1* was broadly expressed in smooth muscle cells, whereas *PIEZO2* expression was scarce (Fig. 1, C and D). Single-cell sequencing data from laboring term myometrium confirmed these observations (23) and further indicated that *PIEZO2* expression was restricted to lymphatic endothelial cells (Fig. S1, J and K). Taken together, these findings reveal a remarkable similarity between human and mouse tissues, with widespread PIEZO1 expression and limited PIEZO2 expression.

The reproductive tract receives sensory innervation that may detect mechanical forces such as stretch and pressure within the uterus and cervix (24). Since PIEZO2 is abundantly expressed in dorsal root ganglia (DRG) and plays a prominent role in somatosensation and interoception (25), we investigated whether it contributes to mechanosensation in the reproductive tract. We labeled *Piezo2*^*+*^ sensory neurons by intrathecally delivering adeno-associated virus (AAV) expressing a Cre-dependent fluorescent protein into *Piezo2*-ires-Cre animals (*Piezo2*^*Cre*^; intrathecal^mScarlet^). This method efficiently labeled DRGs below the T13 vertebral level. Light sheet microscopy revealed that *Piezo2*^+^ sensory fibers primarily innervate the lower reproductive tract (Fig. 1E; Movie S1). High-resolution confocal imaging further showed abundant *Piezo2*^+^ fibers in the vagina and lower uterus, especially near the cervix (Fig. 1F). In contrast, only sparse *Piezo2*^+^ fibers extended into the uterine horn along the broad ligament vasculature, and the myometrium exhibited almost no labeling (Fig. 1F). Given that nerve fiber density in the uterine horn dramatically decreases during late gestation (26, 27), it is expected that *Piezo2*-dependent sensory afferents would originate almost exclusively from the lower reproductive tract at late pregnancy. Moreover, retrograde labeling with cholera toxin subunit B (CTB) revealed minimal overlap with *Sst*, a marker for *Piezo1*^+^ neurons (28). Among 240 CTB-labeled neurons from 4 animals (Fig. S1L), no overlap was observed, indicating that *Piezo1*^+^ innervation is rare in reproductive tissues. Taken together, these findings indicate that *Piezo1* is predominantly expressed within reproductive tissues, whereas *Piezo2* primarily marks the sensory afferents.

### *Piezo1* and *Piezo2* knockout mice have impaired parturition but not gestation

To investigate the functional roles of PIEZO channels during pregnancy and parturition, we generated mice with *Piezo1/2* deletion using *HoxB8*^*Cre*^, broadly targeting tissues caudal to the diaphragm. *HoxB8*^*Cre*^ covered most cells in the uterus except for the epithelium as indicated by the Sun1 reporter in non-pregnant animals (Fig. S2A). We further examined the knockout efficiency in uterine horns at GD18.5 by smFISH. *Piezo1* expression was significantly reduced (Mann-Whitney test p-value<0.0001) in *SMA*+ (smooth muscle actin) smooth muscle cells within the myometrium of the knockout animals (*HoxB8*^*Cre*^; *Piezo2* ^*f/f*^; *Piezo2*^*f/f*^, referred to as HoxB8; P1; P2) whereas its expression in adjacent non-smooth muscle cells, which are primarily lymphatic endothelial cells, remained largely unchanged (Fig. S2, B and C).

**Figure 2.**
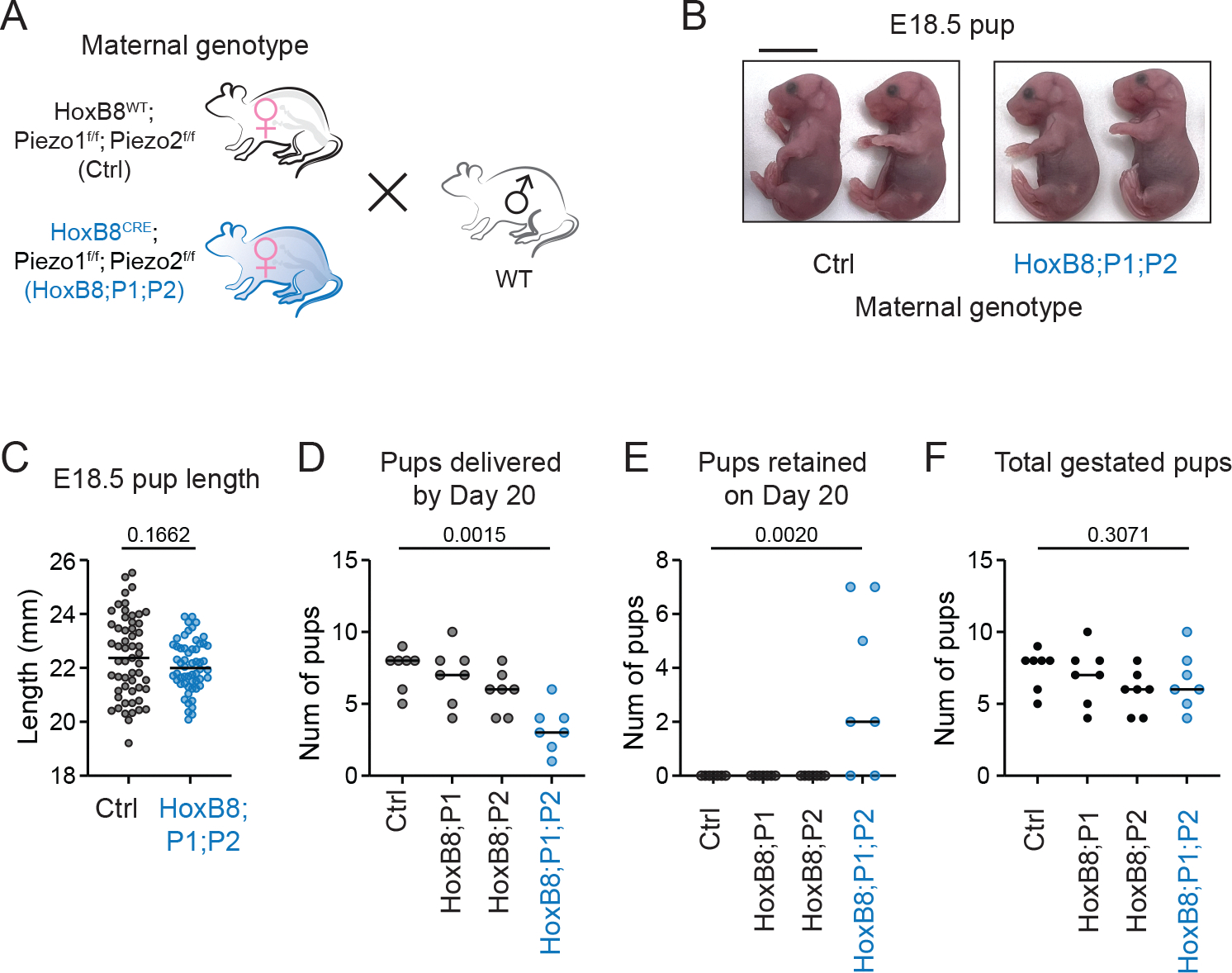
*Piezo1/2* knockout animals have apparently normal gestation but parturition defects. (A) *HoxB8* ^*Cre*^ was used to knock out *Piezo1* and *Piezo2* in most cells relevant to pregnancy. The female animals were mated to wild type males to ensure at least one functional copy of *Piezo1/2* in offsprings. (B) Appearance of embryos at gestational day 18.5. Scale bar: 1 cm. (C) Dimensions of embryos as measured by crown-rump length were similar between control and knockout animals (Welch’s t-test p-value=0.1662). n=55, 56 pups from control and knockout dams, respectively. (D) Fewer pups were delivered by *Piezo1*/2 double knockout animals by GD 20 (Kruskal-Wallis test p-value=0.0028, pairwise comparison between double knockout and ctrl p-value=0.0015), as some animals still had pups retained on GD 20 (Kruskal-Wallis test p-value=0.0006, pairwise comparison between double knockout and ctrl p-value=0.0020) as shown in (E). The total number of pups in gestation was similar regardless of the genotype (Kruskal-Wallis test p-value=0.3071) as in (F). Deletion of *Piezo1* or *Piezo2* alone did not affect the number of pups delivered by day 20. Each point represents one pregnancy from a nulliparous dam. n=7 animals for each group.

To avoid potential complications from knocking out PIEZOs in the developing fetuses, we set up the breeding using wildtype C57BL6/J males so that all embryos in gestation maintained at least one copy of functional *Piezo1/2* (Fig. 2A). As previously reported, *HoxB8*^*Cre*^ mediated *Piezo2* knockout exhibited impaired mating behavior, failing to complete intercourse during the standard single-night mating protocol (29). Nonetheless, continuous mating attempts eventually resulted in successful pregnancies. The overall appearance and size of the embryos in gestation were comparable to Cre- control littermates (Fig. 2, B and C), indicating minimal involvement of PIEZO1/2 in gestation.

At parturition, however, notable complications were observed in *Piezo1/2* double-knockout females, characterized by prolonged labor and incomplete delivery by gestational day 20 (GD20) (Fig. 2, D to F). All animals started parturition on GD19, but retained embryos were still present in many double knockout animals by GD20. The total number of embryos in gestation was similar across genotypes, suggesting that *Piezo1/2* did not affect general fertility but had a specific effect on parturition. Single knockouts showed no evident parturition deficits, suggesting compensatory interactions between *Piezo1* and *Piezo2*.

Because complications in parturition are frequently attributed to issues in cervix remodeling and uterine contraction, we performed histological analysis on uterus and cervix sections. Uterine horns at GD18.5 from control and knockout animals revealed typical layered structures in hematoxylin-eosin (H&E) staining (Fig. S2D). Cervix sections revealed no discernible abnormalities in extracellular matrix (ECM) remodeling by trichrome staining (Fig. S2E). The parturition defects are thus less likely due to complications with cervical remodeling.

### Loss of *Piezo1* and *Piezo2* impairs uterine contraction

**Figure 3.**
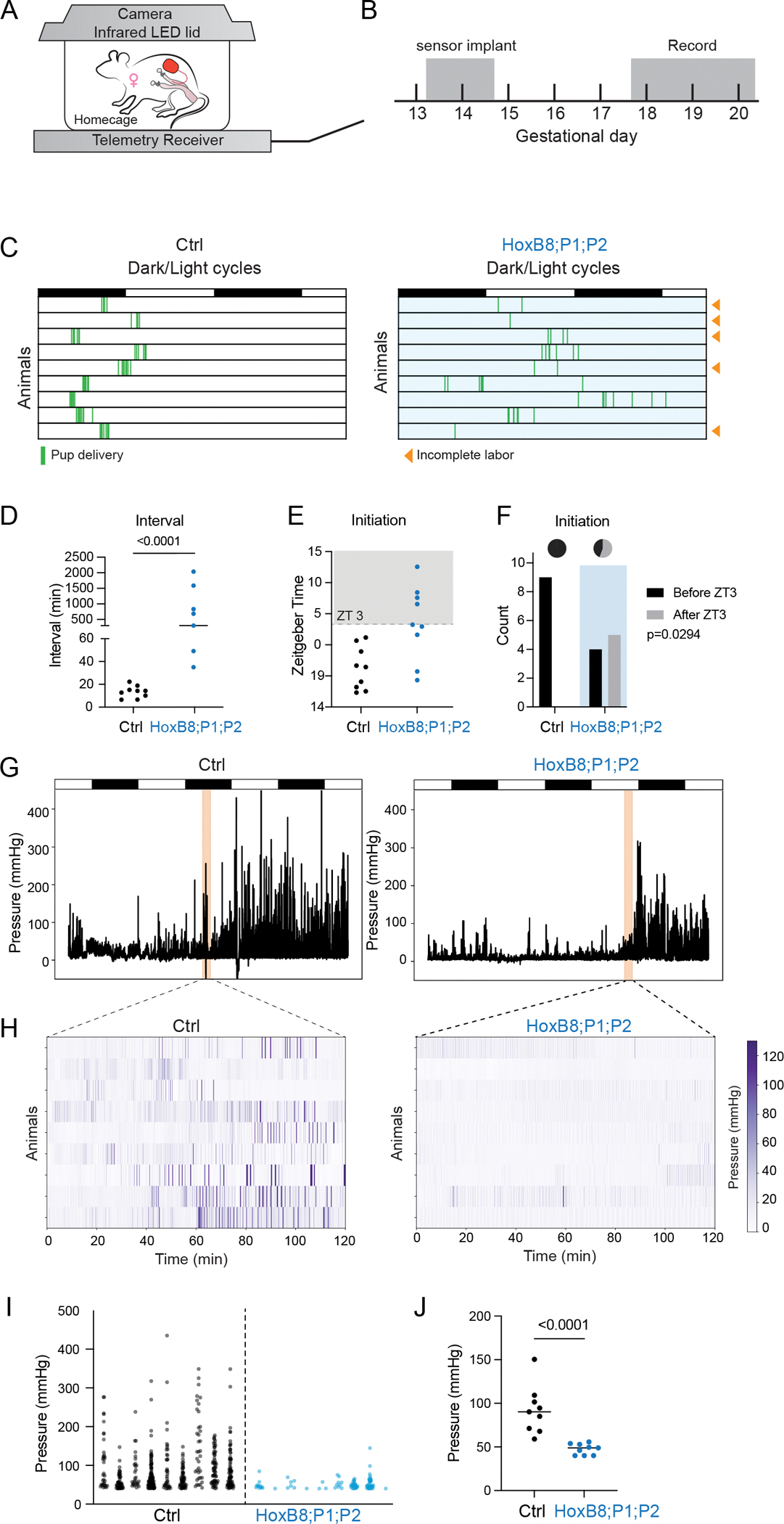
*Piezo1/2* knockout animals have defects in uterine contraction. (A) The recording setup consists of a receiver that records intrauterine pressure signals from the telemetry implants in real time and a custom-made cage lid with infrared LED lights and a camera to record animal activity. (B) Telemetry devices were implanted into the animals at gestational day 13.5–14.5 and the animals were allowed to recover for 3 days. The animals were then recorded continuously during parturition. (C) *HoxB8*^*Cre*^ *Piezo1/2* knockout animals exhibited prolonged labor, and some could not complete delivery at day 20. (D) The average interval between pup delivery (Mann-Whitney test p-value<0.0001). (E) Time of labor onset as marked by appearance of the first pup was delayed in knockout animals. (F) *Piezo1/2* knockout was significantly associated with delayed labor initiation. Fisher’s exact test p-value=0.0294. (G) Representative intrauterine pressure traces from control and knockout animals. (H) Heatmap for intrauterine pressure of all animals in the 2-hour window after labor onset. (I) Contractions detected from intrauterine pressure peaks were reduced in knockout animals. (J) The average pressure for contraction peaks was significantly lower in knockout animals (Mann-Whitney test p-value<0.0001). Data quantified from 9 animals in each group.

To directly assess uterine contractions, we used implantable pressure sensors to continuously measure intrauterine pressure in free-moving animals during labor. We also used a three-dimensional (3D)-printed cage lid equipped with infrared light-emitting diode (LED) lights and camera to monitor animals in their home cages and assess parturition events (Fig. 3, A and B). In this way, we enabled the animals to enter spontaneous labor and obtained data under physiological conditions.

We recorded parturitions from *Piezo1* and *Piezo2* double knockout animals and their control littermates. The labor process in knockout animals was slower, as most control animals completed all deliveries within 2 hours, but the knockout animals delivered over an extended period of time (Fig. 3C). Consequently, the average interval between pups for the knockout animals was significantly longer than control (Fig. 3D. Mann-Whitney test p-value<0.0001), consistent with prolonged labor. The knockout animals also tended to delay the delivery of their first pup, with approximately half delivering after zeitgeber time 3 (ZT3), whereas all control animals initiated delivery before ZT3 (Fig. 3, E and F).

The intrauterine pressure measurements further suggested that the knockout animals had weaker uterine contractions during delivery. Before the onset of parturition intrauterine pressure in both knockout and control animals was overall low, with occasional peaks in intrauterine pressure, but after the delivery of the first pup, control animals had frequent high pressure peaks indicative of strong uterine contractions (Fig. 3G; Fig. S3, A and B). When zoomed into the 2-hour time window after the appearance of the first pup, contractions in the knockout animals were minimal, in contrast to frequent contractions in the control (Fig. 3H). The average pressure of the peaks from the knockout animals was significantly lower compared to the control (Fig. 3, I and J, Mann-Whitney test p-value<0.0001). Nonetheless, most of the knockout animals eventually exhibited compensatory increases in contraction strength (Fig. S3, C and D), suggesting that other mechanisms may still activate strong uterine contractions in the absence of PIEZO1/2. Taken together, these observations suggest that PIEZO channels are critical for robust uterine contractions during labor.

### *Piezo1/2* in the uterus and sensory nerves function complementarily

**Figure 4.**
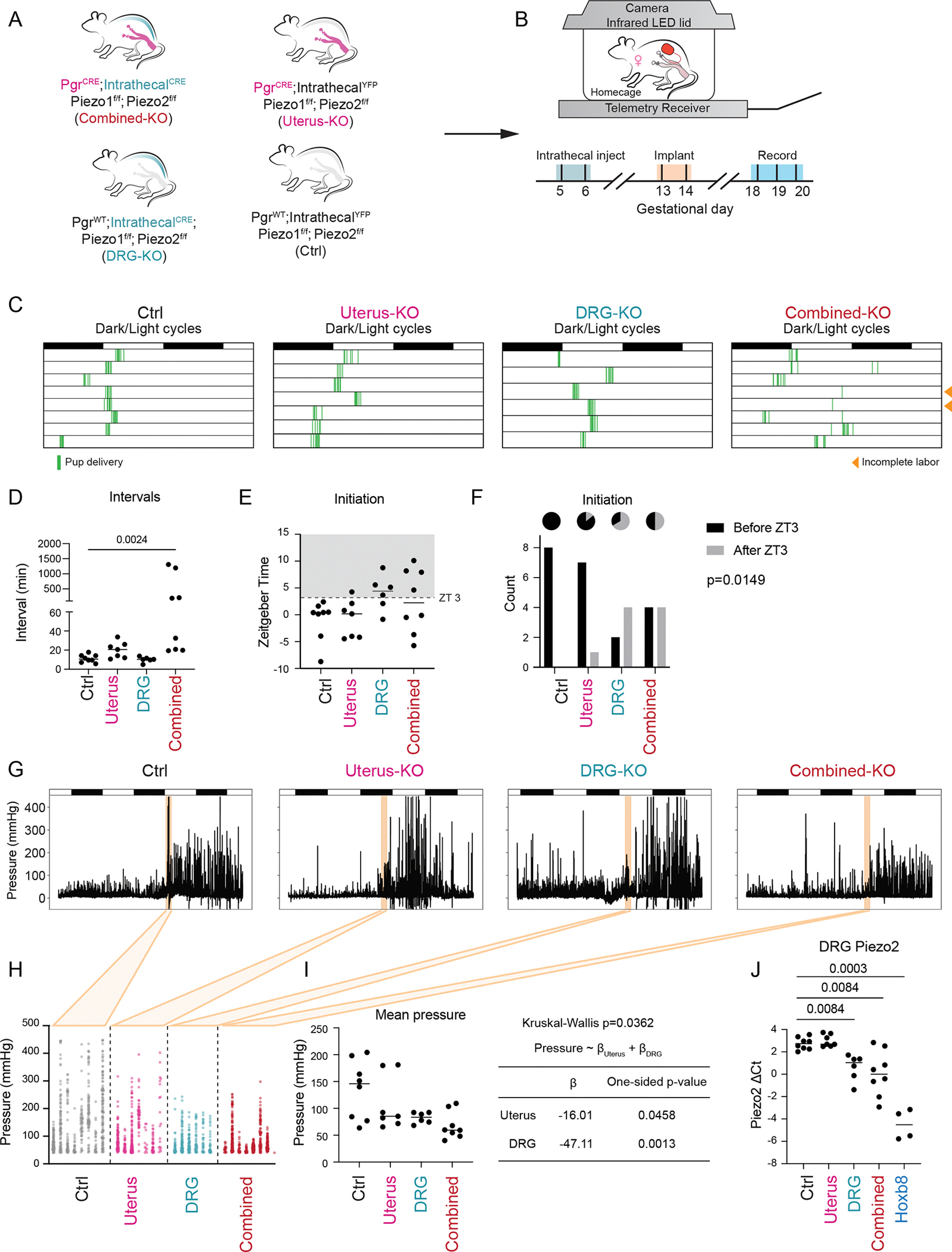
PIEZO in both the uterus and the sensory neurons contribute to labor defects. (A) *Pgr*^*cre*^ was used to target the reproductive tract while MacPNS1 AAV encoding Cre was delivered intrathecally to target the sensory neurons. *Pgr*^*WT*^ animals and AAV encoding YFP were used for corresponding controls. (B) The AAV injection was given at gestational day 5.5–6.5 to avoid affecting mating and pre-implantation events. The telemetry implants were placed at day 13.5–14.5. After 3 days of recovery, the animals were placed under recording for parturition. (C) The animals with *Piezo1/2* deletion in both the uterus and the DRGs had prolonged labor while deletion in the uterus or DRG along had a less severe effect. (D) The mean intervals between pups were significantly increased in combined knockout animals (Kruskal-Wallis test p-value=0.0004, pairwise comparison between combined knockout and ctrl p-value=0.0024). (E) Labor onset was delayed in DRG-specific and combined knockouts. (F) Labor onset after ZT3 was significantly associated with DRG-specific or combined knockouts (Fisher’s exact test p-value=0.0149). (G) Representative intrauterine pressure traces for animals from each group. (H) The pressure peaks within a 2-hour window of delivery for each animal. (I) The mean of peak pressures for each animal differed significantly across groups (Kruskal-Wallis test p-value=0.0362). The effect size from uterus-specific and DRG-specific knockouts was estimated using generalized least square (GLS) model. (J) *Piezo2* expression in L6 and S1 level DRGs was analyzed by qPCR. Animals receiving Cre viruses had significantly lower *Piezo2* expression (Kruskal-Wallis test p-value=0.0001, pairwise q-values=0.0084, 0.0084, and 0.0003 for DRG, combined knockout and *HoxB8* knockout groups in comparison with control group, respectively). Data quantified from 8, 7, 6, 8 animals for control, uterus-specific, DRG-specific and combined knockout groups.

Because *Piezo1* is primarily expressed in reproductive tissues and *Piezo2* in sensory neurons, their apparent compensatory interactions likely arise from distinct mechanisms in these separate compartments. To pinpoint the specific contributions of PIEZO channels in the uterus and sensory neurons, we employed a combinatorial knockout strategy. We generated uterine knockouts using *Pgr*^*Cre*^, which efficiently targets the reproductive tract (10), and confirmed knockout efficiency by smFISH (Fig. S4, A to C). Since Piezo2 is required for respiration, existing sensory neuron-specific Cre lines either cause lethality or only target a limited subset of DRG neurons (30). To overcome these limitations, we delivered AAV expressing Cre intrathecally to target the caudal DRG below the T13 level, a range closely matching that targeted by *HoxB8*^*Cre*^, thus preserving respiratory function while broadly targeting sensory neuron subtypes. This approach enabled us to generate animals with uterus-specific, DRG-specific, and combined knockouts, along with corresponding control groups (Fig. 4, A and B). To avoid complications during mating and early gestation, virus was delivered after embryo implantation (GD 5.5–6.5). Around GD 14.5, the pregnant animals receiving Cre virus began to exhibit signs of proprioceptive defects, consistent with the expected phenotype resulting from PIEZO2 deletion in proprioceptive sensory neurons.

The labor progression showed marked difference between treatment groups (Fig. 4C). Ablation of *Piezo1/2* in the uterus or DRG alone had limited effect on the overall process, whereas combined deletion in both tissues resulted in prolonged labor, resembling the phenotype observed in *HoxB8*^*Cre*^-mediated knockout. Two animals in the combined knockout group even failed to complete labor by noon on GD 20. The average interval between pup deliveries was significantly longer in combined knockouts (Kruskal-Wallis test p-value=0.0024), whereas DRG-specific knockouts exhibited intervals similar to controls (Fig. 4D). Uterus-specific knockouts showed a small increase in the interval but not statistically significant. Furthermore, labor initiation, marked by the appearance of the first pup, was delayed in both DRG and combined knockouts (Fig. 4, E and F). These phenotypic changes closely mirrored those observed for *HoxB8*^*Cre*^-mediated knockout animals, albeit to a lesser extent. Overall, the roles of *Piezo1/2* in DRG neurons and the uterus appear to be additive, with combined deletion leading to more pronounced labor defects.

Intrauterine pressure measurements further supported an additive effect of PIEZO channels from the uterus and DRG neurons (Fig. 4G). Within the 2-hour window following labor onset, uterine contractions were progressively weaker from controls to uterus-specific, DRG-specific, and finally combined knockouts (Fig. 4, H and I). When analyzed using a generalized least squares (GLS) model treating uterus-specific and DRG-specific knockouts as two independent factors, deletion of PIEZO channels in either compartment significantly reduced contraction intensity. The estimated effect size was −16.01 mmHg for uterine knockout (p-value: 0.0458) and −47.11 mmHg for DRG knockout (p-value: 0.0013). Notably, this effect was specific to the 2-hour window after labor onset, as contraction intensity prior to labor (Fig. S4C), or beyond 2 hours after the first pup (Fig. S4D) remained comparable across groups. Moreover, although *Piezo2* expression in caudal DRG (L6 and S1 levels) was significantly reduced in AAV-mediated knockout animals (Kruskal-Wallis test p-value=0.0084), it varied between animals and remained higher than in *HoxB8*^*Cre*^ knockout animals (Fig. 4J), consistent with the milder phenotypes observed in AAV-mediated models. Taken together, our data demonstrate that PIEZO channels in both the reproductive tract and DRG neurons contribute additively to normal labor progression and uterine contractility.

### *PIEZOs are* required for upregulation of gap junctions in the uterus

**Figure 5.**
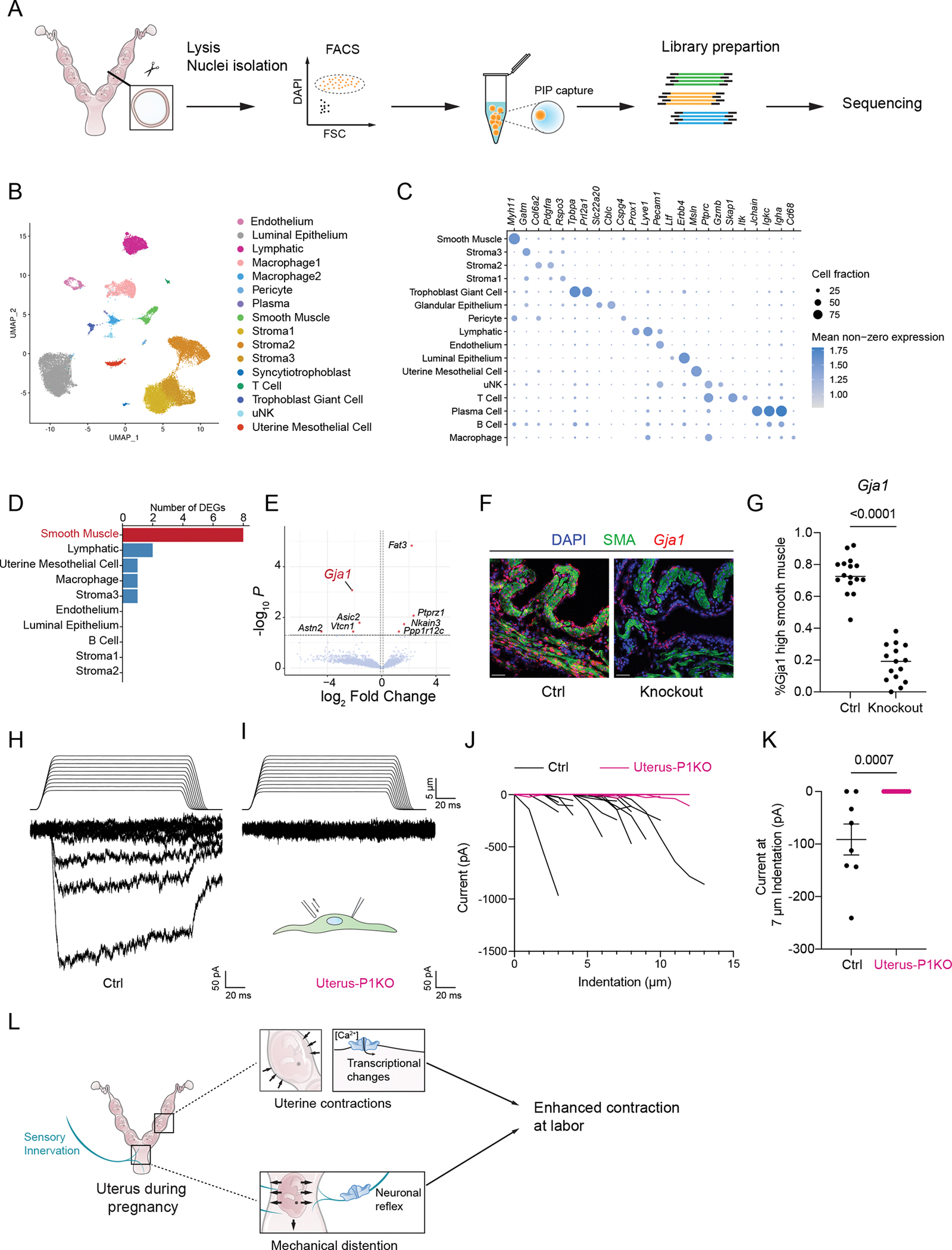
Single nuclei sequencing of the uterus revealed a defect in gap junction upregulation. (A) Uterus samples at gestational day 18.5 were dissected out, isolated from the placenta and the fetus, and processed for single nuclei sequencing. A total of 8 samples were sequenced, including 4 knockout and 4 littermate control animals. (B) Nuclei were clustered into major cell types and some representative cell markers were shown in (C). (D) Differentially expressed genes were identified for major cell types between control and *Piezo1*/2 knockout samples. Most DEGs were within smooth muscle cells. (E) The DEGs in smooth muscle are shown with fold change and adjusted p-value. (F) smFISH of *Gja1* combined with immunofluorescence of smooth muscle actin (SMA) revealed that the *Gja1* expression was reduced in knockouts, as quantified in (G) (Mann-Whitney test p-value<0.0001). Scale bar: 50 μm. Images taken from 3 animals for each group. n=16, 14 sections for control and knockout groups, respectively. Mechanically activated currents were recorded from cultured smooth muscle cells, with representative traces for control (H) and uterus-specific knockout animals (I) (*Pgr*^*Cre*^; *Piezo1* ^f/f^; *Piezo2* ^f/+^) at GD 16.5. (J) Indentation-induced current was diminished in cells from knockout animals, as quantified in (K) (Mann-Whitney test p-value=0.0007). Smooth muscle cells cultured from 3 different animals for each genotype, n=16, 14 cells for control and knockout respectively. (L) The overall role of *Piezo1*/2 in parturition. *Piezo1*/2 in the uterus mediates calcium influx, leading to transcriptional changes that enhance contractility, while sensory innervation mediates neuronal reflexes that promote uterine contraction. The two compartments cooperatively promote parturition.

PIEZO1 and PIEZO2 function as low-threshold mechanosensors that mediate Ca^2+^ influx. Notably, uterine contractions occurring as early as one day before labor generate pressures exceeding the half-maximal activation threshold (P50) of PIEZO1 (−28.0 ± 1.8 mm Hg (31)), suggesting that these channels are likely activated well before labor onset. Such early activation is poised to not only transiently alter intracellular calcium dynamics but also initiate long-term transcriptional changes. To test this hypothesis and identify PIEZO-dependent transcriptional changes in the uterus, we performed single-nuclei RNA sequencing (snRNA-seq) on uterine horns from GD18.5 knockout and control animals (*HoxB8*; P1; P2 or control littermates) (Fig. 5A; Fig. S5A).

After clustering nuclei based on their transcriptomes, we visualized the data in a 2D plot generated by Uniform Manifold Approximation and Projection (UMAP) (Fig. 5B; Fig. S5B). Major uterine cell types were well separated in the plots and expressed their corresponding marker genes (Fig. 5C). Nuclei from different animals or different genotypes showed substantial overlap in the UMAP plots (Fig. S5, C and D), suggesting that *Piezo1/2* deletion did not disrupt uterine development. This observation aligns with our earlier findings that knockout animals experience normal gestation and is further supported by cell proportion analysis (Fig. S5E), which revealed similar fractions of all cell types in control and knockout uterus samples.

We next investigated differentially expressed genes (DEGs) in each cell type. As expected for PIEZOs as ion channels that influence transcription indirectly through cumulative cation influx, only a small number of DEGs were identified (Fig. 5D). Most of the DEGs were detected in smooth muscle cells, the principal mediators of uterine contractions in labor. These genes may be regulated by intracellular Ca^2+^ dynamics and are thus sensitive to the loss of PIEZO activity. Among the DEGs, gap junction protein α1 (*Gja1*, also known as connexin 43), specifically induced late in gestation (32), was significantly reduced in smooth muscle cells from knockout animals (Fig. 5E, adjusted p-value=0.0008). Notably, previous studies have shown that smooth muscle-specific *Gja1* knockout mice exhibit delayed, and occasionally incomplete labor (32), aligning with our observations in *Piezo1/2* double knockout animals. We further validated the results with smFISH. Although *Gja1* was minimally expressed in non-pregnant uteri (Fig. S5F), its expression increased on GD 18.5, with control animals displaying significantly higher levels in smooth muscle cells (Fig. 5, F and G, Mann-Whitney test p-value<0.0001). Similar reduction was also observed for uterus-specific knockout (Fig. S5, G and H). Furthermore, cultured smooth muscle cells from GD 16.5 *Piezo1*-null uteri displayed a significant reduction in mechanically-activated current (Fig. 5, H to K, Mann-Whitney test p=0.0007), establishing PIEZO1 as the primary mechanotransducer. An analogous decrease was also seen in cells from non-pregnant uteri (Fig. S5, I to M). Although the flat morphology of the cells limited the range of applicable indentation depths and hindered the measurement of saturating currents, the pronounced reduction in currents elicited by intermediate indentations confirms PIEZO1-dependency of these responses. These findings suggest that PIEZO channels in the uterus play a critical role in promoting *Gja1* upregulation before labor, and that reduced *Gja1* expression likely contributes to impaired uterine contractions in the PIEZO knockouts.

## Discussion

PIEZO1 and PIEZO2 have been established as the main mechanosensitive channels in multiple cell types. In smooth muscle cells, PIEZO1 acts as the primary mechanosensitive receptor that mediates blood pressure-dependent remodeling, and genetic deletion of PIEZO1 abolishes stretch-activated currents completely (33). Similarly, PIEZO2 functions in sensory neurons to detect tactile pressure (34), bladder volume (35), gastrointestinal stretch (36), and other mechanical stimuli, and its ablation silences mechanical responses in corresponding neurons (35–37).

Building on these foundational roles, our study explored the function of PIEZO channels in reproductive physiology, revealing their regulation of parturition through overlapping, yet distinct, mechanosensory pathways (Fig. 5L). We found that while individual deletion of PIEZO1 or PIEZO2 produced only mild effects, simultaneous knockout of both channels resulted in severe defects, suggesting that PIEZO1 and PIEZO2 have complementary functions, with each partially compensating for the other during parturition. As a result of the lack of a reliable detection method for endogenous PIEZO protein, we assessed changes in PIEZO transcripts as a proxy for channel expression. Although transcript abundance may not directly reflect channel activity, it offers insights into the spatial expression patterns and relative changes in knockout animals.

Mechanotransduction during parturition operates in at least two key domains: the reproductive tract and its sensory innervation. In the reproductive tract, uterine contractions generate mechanical forces that activate PIEZO channels, a process that may begin well before labor onset. Consistent with a role in early mechanical sensing, we observed PIEZO-dependent upregulation of *Gja1*, which encodes connexin 43, a protein crucial for coordinating uterine contractions. Previous studies have shown that transcription of *Gja1* is induced by mechanical stretch but suppressed by progesterone signaling; this suppression is lifted at late gestation, permitting its transcription (38). Moreover, calcium influx through PIEZO channels can activate phospholipase C (PLC), NF-κB, and other transcription factors, many of which drive transcription of contractility genes in the myometrium (39, 40). Thus, calcium influx through PIEZOs may facilitate translating mechanical stretch into transcriptional regulation of contractility-associated genes. Interestingly, the persistence of *Gja1* transcripts–albeit at markedly reduced levels–in the GD18.5 myometrium of *Piezo1/2* knockouts suggests that PIEZO-independent mechanisms can still partially activate expression of contractility-associated genes. In addition, because gap junction function is also regulated translationally and post-translationally (41–43), transcript changes may not linearly predict coupling strength; direct functional assays are needed to assess the extent to which PIEZO knockout affects gap junction activity.

In parallel, sensory innervation of the reproductive tract, especially the cervix and vagina, constitutes a second mechanosensory mechanism during parturition. We hypothesize that mechanical distension of these tissues as the fetus descends activates PIEZO2-expressing primary afferents, initiating the Ferguson reflex, a neuroendocrine circuit that stimulates hypothalamic oxytocin release, strengthens uterine contractions, and promotes labor in multiple mammalian species, including humans (12, 44–50). Given that PIEZO2 is essential for tactile and visceral mechanosensation in diverse contexts (29, 30, 34–36), it is well positioned to serve as the primary sensor that triggers this reflex. Validation of this hypothesis will require time-resolved oxytocin measurements in laboring *Piezo2*-deficient animals. Serial blood sampling is impractical in mice given their limited circulating volume, but in vivo recording using genetically encoded sensors may be a feasible alternative (51). Beyond oxytocin release, sensory afferents transmit labor pain, which can elevate stress hormones, including endorphins, catecholamines, and cortisol, and may prolong labor when present at excessive levels (52–55). Under physiological conditions, PIEZO2 is the principal transducer in low-threshold mechanoreceptors, whereas only a small subset of nociceptors expresses functional PIEZO2 (56). Inflammation can potentiate PIEZO2 currents and produce allodynia (57–59); such up-regulation is plausible in the cervix, where remodeling is accompanied by inflammation as labor approaches. Despite these opposing effects, sensory-specific *Piezo2* deletion delays parturition, suggesting that PIEZO2-driven oxytocin release and mechanosensory feedback might outweigh any pain-mediated slowing of labor.

Our study also underscores the extensive complexity of mechanotransduction during parturition. While our experiments using *Pgr*^*Cre*^-mediated uterine knockout combined with AAV-mediated DRG knockout produced parturition phenotypes similar to those seen in *HoxB8*^*Cre*^ knockouts, the latter exhibited more severe labor defects. This difference may be partially attributed to lower knockout efficiency achieved with viral delivery, but it is also possible that additional cell types targeted by *HoxB8*^*Cre*^ were not affected by our combined knockout strategy.

Additional complexity also exists within the reproductive tract and sensory innervation, the two primary compartments we focused on. Within the reproductive tract, our models did not target all cell types, such as endothelial cells (both vascular and lymphatic), which are critical for gestation (60) and known to engage in PIEZO-dependent signaling (5). Similarly, sensory innervation includes many calcitonin gene-related peptide (CGRP)-subtype sensory fibers, known to be polymodal and capable of integrating sensory inputs from multiple receptors (56). A subset of CGRP neurons can respond to high-threshold mechanical stimuli independently of PIEZO2 (56), suggesting the presence of alternative mechanosensitive pathways.

Moreover, loss of PIEZO1 and PIEZO2 did not eliminate all mechanical force-associated changes during pregnancy, including uterine hypertrophy and cervical remodeling. These observations suggest that additional mechanosensitive pathways contribute to the regulation of pregnancy and parturition, especially during early pregnancy. Indeed, TRP channels (18, 20), Hippo/YAP signaling (16), MAPK signaling (14) and several other pathways can also respond to certain mechanical forces and mediate key adaptations in pregnancy. Building a comprehensive network that integrates these mechanosensitive pathways with PIEZO-mediated processes will be a critical direction for future research.

While our study demonstrates that PIEZO channels are crucial for parturition in mice, direct validation of our findings in humans remains challenging due to severe physiological consequences associated with PIEZO loss-of-function mutations. PIEZO1 knockout mice exhibit embryonic lethality (61), and PIEZO2 deficiency in humans results in profound deficits in proprioception, tactile sensation and interoception (62). However, accumulating evidence suggests that mechanotransduction pathways are conserved across species. PIEZO1 and PIEZO2 exhibit conserved expression patterns in both mouse and human myometrium, and GJA1 remains the predominant connexin protein expressed in human myometrium (63). Moreover, calcium signaling is universally central to uterine contractions across mammalian species. Clinically, nifedipine, a commonly used tocolytic drug, exerts its effect by inhibiting voltage-gated L-type calcium channels (64). Given the conserved expression patterns of PIEZO1 and PIEZO2 in mouse and human myometrium, along with the universal importance of calcium signaling in uterine contractions, it is plausible that PIEZO channels function cooperatively with L-type calcium channels, raising the possibility that pharmacological inhibition of PIEZO channels could enhance the efficacy of existing tocolytic therapies. Physical or pharmacological activation of PIEZO might in turn facilitate labor induction.

## Materials and methods

### Animals

Mice were group-housed in standard housing with 12:12h light:dark (light on at 6 am, off at 6 pm) with ad libitum access to chow diet and water unless specified. Light-on time is defined as zeitgeber time (ZT) 0. Room temperature was maintained at approximately 22 °C and humidity between 30–80% (not controlled). Mice for telemetry recordings were single housed. Mice 8–12 weeks of age from the following strains were used for this study: wild-type (WT) C57BL/6J (Jackson #000664), *Piezo2*^Cre^ (*Piezo2*^tm1.1(cre)Apat^, Jackson #027719) (65), *HoxB8*^Cre^ (MGI:4881836) (66), *Pgr*^Cre^ (Jackson *Pgr*^tm1.1(cre)Shah^/AndJ, Jackson #017915) (67), *Piezo2*^fl/fl^ (*Piezo2*^tm2.2Apat^/J, Jackson #027720) (34) and *Piezo1*^fl/fl^ (*Piezo1*^tm2.1Apat^/J, Jackson #029213) (68). All animals were maintained on C57BL/6J background, except for *Piezo2*^Cre^, which were on CD-1;C57BL/6J background. Only female animals were used for pregnancy-related studies. During mating, each female mouse was housed with a WT male after 4 pm (ZT10). Pregnancy was confirmed by identification of a semen plug before 8 am (ZT2) and that morning was recorded as gestational day 0.5 (GD0.5). All animal use protocols were approved by The Scripps Research Institute Institutional Animal Care and Use Committee and were in accordance with the guidelines from the NIH.

### Human Myometrial Samples

De-identified human myometrial tissues were obtained from the Reproductive Specimen Processing and Banking Biorepository at Washington University in St. Louis. Inclusion criteria for samples used in this study were gestational age ≥37 weeks, singleton gestation, and nulliparity. Additional exclusion criteria were known HIV, hepatitis B or hepatitis C infection. Human myometrial biopsies were collected from the lower uterine segment of the hysterotomy performed at the time of scheduled cesarian delivery, prior to labor. Samples were transported on ice, flash frozen in liquid nitrogen within 60 minutes of collection and stored at −80 °C until further analyses. Use of human samples was under the approval of the Scripps Research Institute Institutional Review Boards (IRB-23–8255).

### Adeno-Associated Viruses (AAVs)

MacPNS.1-CAG-DIO-mScarlet (capsid Addgene #185136 (69)) was used to label *Piezo2*^+^ sensory neurons. MacPNS.1-CAG-iCre-YFP, MacPNS.1-CAG-YFP were used to generate DRG-specific knockouts and control groups for functional studies. The AAV cargo vectors were cloned, and packaged into AAVs in-house following a published protocol (70). After titration by qPCR, AAV was aliquoted in 10 μL volumes and flash frozen for long-term storage.

### Surgeries

Isoflurane (4% for induction, 1.5–2% for maintenance) was used for general anesthesia. The surgery region was shaved, cleaned and sterilized with ethanol and povidone-iodine. Post-operatively, mice received a subcutaneous injection of flunixin and topical antibiotic ointment for pain management and infection prevention.

The telemetry device used was HD-X11 from Data Science International (DSI) and the surgery followed previously published procedure (71). To implant telemetry devices, an incision along the midline was made to each animal at GD13.5–14.5 and the uterus was gently retracted. At least 3 embryos in gestation needed to be present on one side of the uterine horn for implantation. A small incision just large enough for the entry of the pressure catheter was made in the uterine horn at a region between embryos and the pressure catheter from the device was inserted past 1.5 embryos. The catheter was then secured in place by adding 1 μL of tissue adhesive at the site of entry. If necessary, the uterus was closed with a single suture using 8–0 nylon suture. The abdominal incision was closed with sutures afterwards. For prophylaxis of infection, sulfamethoxazole and trimethoprim (1:1000 dilution of Equisul-SDT, 400 μg/ml total active ingredients) were added to the drinking water one day before and two days after the surgery.

For CTB retrograde labeling, each animal received 4–5 μL of 0.1% CTB-488 (Invitrogen C34775) or CTB-647 (Invitrogen C34778) in PBS. The injections were made with glass pipettes mounted onto Hamilton syringes and delivered 0.2–0.5 μL per site. Multiple injections were made throughout the entire uterus/vagina. DRGs were harvested 3–4 days post-surgery for histology.

For intrathecal injection, 8–10 μL of AAV was delivered at L5-L6 junction using a Hamilton syringe. For sensory nerve labeling, MacPNS.1 CAG-DIO-mScarlet AAV was injected into *Piezo2*^*Cre*^ mice at a total dose of 6E11 vg. The tissues were collected 3–4 weeks after injection. For DRG-specific knockouts in functional assays, MacPNS.1 CAG-YFP or MacPNS.1 CAG-iCre-YFP was delivered into animals at GD5.5–6.5 with a total dose of 3E11 vg.

### Preparation of single nuclei sequencing

Animals were euthanized at 10–11 am on GD18.5 under deep anesthesia with isoflurane and the uterus was dissected out and placed in ice-cold PBS. The fetus along with placenta and amniotic membranes was gently peeled off and the uterus was blotted dry on a Kimi wipe before being frozen in liquid nitrogen. On the day of sequencing sample preparation, the frozen samples were pulverized with a pestle chilled with liquid nitrogen. All buffers used in later steps contain 0.5 U/ml RNase inhibitor (3335399001, Sigma-Aldrich). The tissues were then resuspended in lysis buffer (21mM MgCl_2_, 1mM CaCl_2_, 146mM NaCl, 10mM Tris pH 8, 0.02% Tween-20, 1% Polyvinylpyrrolidone) and lysed in a pre-chilled dounce homogenizer. Homogenized tissue was passed through 70 μm cell strainer to remove large debris and then spun down at 500 g for 5 min. Nuclei were resuspended in wash buffer (1mM MgCl_2_, 1mM CaCl_2_, 146mM NaCl, 10mM Tris pH 8, 1% Polyvinylpyrrolidone) containing 5 μg/ml DAPI before loading onto Sony MA900 Sorter equipped with a 100-μm microfluidic sorting chip. Single nuclei were sorted into a collection tube and then centrifuged at 500 g, 5 min at 4 C. After resuspension in NSB (Fluent BioSciences) and then counted using a hemocytometer, 5000 nuclei were taken for PIPseq library preparation using the PIPseq T2 v4.0PLUS 3’ kit (Fluent BioSciences) following the manufacturer’s instructions (72). The library was sequenced on Element Aviti sequencer following the Adept Workflow.

### Tissue clearing and staining

Tissues were cleared for imaging following the HYBRiD clearing protocol (73). In brief, the reproductive tract was dissected out after trans-cardial perfusion of ice-cold PBS and then 4% PFA (Electron Microscopy Sciences, 15714). The samples were then subjected to organic clearing by THF/25% Quadrol gradient and DCM. After embedding into A1P4 hydrogel (1% acrylamide, 0.125% Bis, 4% PFA, 0.025% VA-044 (w/v), in 1x PBS), the samples were then cleared with LiOH-Boric-SDS buffer until translucent.

The tissues were washed extensively in PBS before immunolabeling. The tissues were incubated in Rabbit anti-RFP antibody (Rockland #600–401-379, 1:500) in PBST (1 x PBS, 0.1% Tween-20, 0.1% Triton X-100) for 5 days at RT and then washed with PBST. This was followed by incubation with Donkey anti-Rabbit-AlexaFlour PLUS 647 antibody (ThermoFisher, A32795, 1:1000) for 5 days at RT, and then sequentially washed in PBST. The samples were then equilibrated in EasyIndex (RI 1.52, LifeCanvas) for confocal and light sheet microscopy imaging.

### RNA Fluorescence in Situ Hybridization (RNAscope)

Mice were deeply anesthetized by isoflurane prior to perfusion with 20–25 ml ice-cold PBS. Tissues were dissected and then fixed in ice-cold 4% PFA for 1–2 hr, before transferring to 30% sucrose for dehydration overnight. The tissues were then embedded in Tissue-Tek OCT Compound (Sakura Finetek). The uterus samples were cryosectioned at 15 μm and collected onto SuperFrost Plus Microscope Slides (Fisher Scientific 12–550-15). The target transcripts were detected with RNAscope Multiplex Fluorescent Reagent Kit v2 Assay (Advanced Cell Diagnostics) following the manufacturer’s protocol for fixed-frozen tissues. Human tissue samples were directly embedded for cryosection and then stained according to the manufacturer’s protocol for fresh-frozen tissues. The following probes were used: Mm-*Piezo2*-E43-E45 (Advanced Cell Diagnostics 439971), Mm-*Piezo1*-O1 (500511, Advanced Cell Diagnostics), Mm-*Gja1* (Advanced Cell Diagnostics 486191) for mouse tissues, and Hs-*PIEZO1* (Advanced Cell Diagnostics 485101), Hs-*PIEZO2* (Advanced Cell Diagnostics 449951) for human tissues. Cell markers were stained with antibody afterwards for smooth muscle actin (*Acta2*, Abcam ab267536) or *Lyve1* (R&D systems AF2125). Sections were mounted using Prolong Glass Antifade Reagent (ThermoFisher P36982) and imaged under a 20X objective on a Nikon AX confocal microscope.

### Tissue histology

Mice were deeply anesthetized by isoflurane prior to perfusion with 20–25 ml ice-cold PBS. Tissues were dissected and then fixed in ice-cold 4% PFA overnight and then dehydrated in 70% ethanol. The tissues were then sent for paraffin embedding, sectioning and staining at the Sanford Burnham Prebys Histology Core. The stained slides were imaged with a Leica Asperio AT2 slide scanner.

### Confocal microscopy

Mounted uterus samples were imaged with Olympus FV3000 confocal microscope using the following objectives: 4X, 0.28 NA, air (XLFluor, Olympus); 10X, 0.6 NA, water immersion (XLUMPlanFI, Olympus). Images were acquired using Fluoview software (v2.4.1.198).

RNAscope samples and DRG samples were imaged with Nikon AX confocal microscope with a 20x objective or a 16x water immersion objective.

### Light sheet microscopy

The uterus samples were mounted using 1% agarose/EasyIndex. The samples were equilibrated in SmartSPIM chamber filled with EasyIndex Matched Immersion Oil (LifeCanvas) overnight before imaging. Images were acquired using a 3.6X, 0.2 NA objective (LifeCanvas) with bilateral illumination along the central plane of symmetry within the sample. The voxel size after stitching was 1.8/1.8/2 μm.

### Mouse imaging

Mice were imaged using a SainSmart 5MP 1080p Night Vision camera module with a 160° field of view (FOV) fisheye lens to capture a large field of view. Two infrared red lights were installed on both sides of the camera to enable imaging in the dark. Raspberry Pi Zero W was used to control the camera and store the recorded videos. MotioneyeOS was used to remotely control the recordings and minimize disturbance to the animals. The camera and Raspberry Pi were housed in 3D printed capsules made with Nylon X using a Fusion 3, F410 high-performance 3D printer. The camera and Raspberry Pi capsules were printed using electrostatic discharges (ESD)-safe PLA 3D printing filament (3DXSTAT) to prevent ESD. The design files and relevant code have been deposited on Zenodo (74).

### RNA extraction

DRG were dissected out and flash frozen in liquid nitrogen. Total RNA was extracted from frozen tissue using TRIzol (ThermoFisher 15596018) and RNeasy Micro Kits (Qiagen 74004) following manufacturer’s instructions.

### Reverse Transcription-PCR

For RT-PCR analysis, total RNA was reverse-transcribed with Maxima H Minus First Strand cDNA Synthesis Kit (Thermo Fisher K1652). PCR reaction was set up with SsoAdvanced Universal SYBR Green Supermix (Bio-Rad) on CFX384 real-time PCR system (Bio-Rad). Normalized mRNA expression was calculated using ΔCt method, using *Tbp* (encoding TATA-box-binding protein) mRNA as the reference gene. Statistical analysis was performed on Ct values. Primer sequences (forward and reverse sequence, 5’ → 3’, respectively) are *Tbp* (CCTTGTACCCTTCACCAATGAC and ACAGCCAAGATTCACGGTAGA); *Piezo2* (CAAAGTCAATGGTCGCGTGT and CAAGGCTGGCCGTCATATTC).

### Electrophysiological recordings

Smooth muscle cells from *Piezo1* knockout (*Pgr*^Cre^; *Piezo1* ^f/f^; *Piezo2* ^f/+^) animals and their Cre-negative littermates were isolated following a published protocol (75, 76). Briefly, non-pregnant animals in estrus, or pregnant animals at gestational day 16.5 were deeply anesthetized under isoflurane and then perfused with 20–25 mL ice-cold PBS. The uterine horns were dissected out and opened longitudinally along the broad ligament. The stromal layer was mechanically separated with No. 5 Dumont forceps, and the myometrium layer was transferred into HBSS containing 0.15 mg/mL collagenase I (ThermoFisher 17100017). After 35 min incubation at 37 °C, the tissues were washed 3 times in HBSS before being transferred into HBSS with 0.5% trypsin (ThermoFisher 27250018) and 5 mg/ml collagenase I. After incubation at 37 °C for 1 hr, digestion was halted with 10% charcoal stripped FBS (ThermoFisher 12676029) and tissues were lysed by gently pipetting and then filtered through 70 μm nylon mesh. The cells were centrifuged at 400 g for 5 min and resuspended in smooth muscle medium (Vascular cell basal medium (ATCC PCS-100–030), supplemented with growth factors from Vascular smooth muscle cell growth kit( ATCC PCS-100–042, 5 ng/mL rh bFGF, 5 μg/mL rh Insulin, rh EGF 5 ng/ml, ascorbic acid 50 μg/mL), 1x Penicillin-Streptomycin-Glutamine (ThermoFisher 10378016), 10% charcoal-stripped FBS, 50 μg/mL gentamycin (ThermoFisher 15710064)). The cells were plated on gelatin-coated tissue culture dishes and the medium was replaced 2 h after plating to keep only adherent cells. 1–2 days after plating, the cells were digested with 0.05% trypsin and replated onto poly-D-lysine coated glass coverslips (Corning Cat#354086 or VWR Cat#GG-12-PDL) that had been coated with 0.2 mg/mL Matrigel (Corning Cat#354234) for 1h at 37 °C. Most cells (>90%) at this stage were positive for smooth muscle actin and were smooth muscle cells. Uterine smooth muscle cells were identified by their flat, typical muscle cell-like morphology. Patch pipettes were pulled from borosilicate glass capillaries (Sutter, Cat#BF150–86-10) using a P-97 Flaming/Brown micropipette puller (Sutter Instruments) and had a range of resistances from 4–8 MΩ when filled with the intracellular recording solution (see below). To access mechanically-evoked current, the plasma membrane of patched cells was voltage-clamped at the holding potential of –80 mV at the whole-cell configuration. The cells were mechanically stimulated by indentations using a glass probe polished to a 3 to 4 μm diameter (Sutter Cat#B150–110-10). The probe was advanced with a ramp speed of 1 μm/ms and held at the indicated position for 125 ms, driven by a piezoelectric controller and actuator (Physik Instrumente, E-625.SR Compact LVPZT controller/amplifier and P-601.1SL PiezoMove^®^ nanopositioning actuator) attached to the micromanipulator with a custom dye-anodized aluminum adapter at an 80° angle to the cell being recorded. All recordings were performed using a Multiclamp 700A amplifier, Digidata 1550B, and pClamp10.7 (all from Molecular Devices). Extracellular recording solution contained: 135 mM NaCl, 3 mM KCl, 1 mM MgCl2, 2.5 mM CaCl2, 10 mM D-glucose, 10 mM HEPES (pH 7.3 with NaOH; 300–310 mOsm/kg adjusted with D-mannitol to be ~10 mOsm higher than intracellular solution). Intracellular recording solution contained: 133 mM CsCl, 5 mM EGTA, 1 mM MgCl2, 1 mM CaCl2, 10 mM HEPES, 4 mM Mg·ATP, 0.4 mM Na2GTP (pH 7.3 with CsOH; 285–300 mOsm/kg). Currents were sampled at 20 kHz and filtered at 10 kHz. All recordings were conducted at ambient temperature.

Data was analyzed using Clampfit 10.7 and GraphPad Prism v.10.1.2 (DotMatics). The mechanosensitive current was quantified by measuring the peak amplitude of the current recorded during mechanical stimulation, after subtracting the baseline current recorded prior to the stimulation. The baseline current for each trace was estimated as the mean current measured between 35 milliseconds and 5 milliseconds before the onset of mechanical stimulation. A current was classified as a mechanoresponse only if its amplitude exceeded five times the standard deviation of the mean baseline current. Prior to analysis, recordings were low-pass filtered at 500 Hz (with a −3 dB cutoff) using an 8-pole Bessel filter.

### Transcript quantification in RNAscope

Maximum z-projection images were used for RNAscope quantification using CellProfiler (77). Cells were identified based on expanded regions from nuclei DAPI staining. Puncta from RNAscope signals were identified as objects and assigned to corresponding cell nuclei. Immunofluorescent signals such as SMA were quantified by intensity and used to differentiate cell types.

### snRNA-seq analysis

The sequencing results were obtained in fastq format. After transcriptome mapping using PIPseeker (Fluent BioSciences), the raw matrix was filtered with CellBender (78) to remove empty droplets and then filtered out nuclei with high mitochondria reads (>5%) using Seurat (79). Doublets were detected and filtered using DoubletFinder (80). Feature selection was performed with *FindVariableFeatures* within Seurat. Data from each sample was scaled and then integrated using Harmony (81). Uniform manifold approximation and projection (UMAP) was used for visualization after integration. Cell types were assigned based on markers from published sequencing projects (22, 23). Cell proportions across different conditions were tested by Propeller (82) for difference. Differentially expressed genes in each cell type were identified using SVA-EdgeR following a previously published procedure (83). For re-analysis of published mouse uterus (22) and human myometrium (23) single cell RNA-seq, the datasets were downloaded and imported into Seurat. Mouse sequencing data was downloaded from Arrayexpress (Accession: E-MTAB-11491, Ind001 to Ind016, processed matrices for uterus samples). Human myometrium data was downloaded from the database of Genotypes and Phenotypes (dbGaP, data accession: phs001885.v5.p1, datasets SRR18927399 to SRR18927405). Access for the human single cell RNA-seq data was approved with Project #35636. Different samples were integrated using Harmony and UMAP was used for visualization after integration. All scripts used for analysis are available through Zenodo (84).

### Telemetry analysis

Intrauterine pressure data was exported in ASCII format from Ponemah 5.20. Pup delivery was manually annotated from video. The time for birth was determined with an accuracy of 1 minute. Pressure data was analyzed with a custom Python package “peakplot” available on Github (85). The telemetry data can be accessed through Zenodo (86). The pressure measurements were resampled to a sampling rate of 1 per second. The pressure peaks with a minimum height of 40 (mmHg) and prominence of 20 were identified using scipy.find_peaks function and statistical tests (Mann-Whitney test or Kruskal-Wallis test) were conducted in GraphPad Prism. To analyze the effect of uterus-specific and DRG-specific knockouts on peak pressures, average peak pressures from animals in the 4 treatment groups (control, uterus-specific, DRG-specific and combined knockouts) were considered in a linear model for the continuous outcome $P_{i}$, the average peak pressure for the i-th animal, which depends on two categorical predictors $D_{\text{DRG}}$ and $D_{\text{Uterus}}$ denoting whether PIEZO1/2 is deleted from DRG or uterus of this animal. For observation i, the model is

$$P_{i}=\beta_{0}+\beta_{DRG}D_{DRGi}+\beta_{\text{Uterus}}D_{\text{Uterus}\;i}+\varepsilon_{i}$$

The error term $\varepsilon_{i}$ is assumed to follow a normal distribution with mean 0 but with group-dependent variance:

$$\varepsilon_{i}∼N\left(0,\sigma_{g_{i}}^{2}\right)$$

Here, $g_{i}$ indicates the treatment group to which observation i belongs. In matrix form, the model can be written as

P=Xβ+ε,ε∼N(0,Ω)

Where P is the vector of outcomes, X is the design matrix including the intercept and the categorical factors, β is the vector of parameters, and Ω is a diagonal covariance matrix for the error terms.

The variances are not equal across the groups (heteroscedasticity) as viral-induced knockouts are inherently more variable than Cre expressed from a genomic locus. We thus used Generalized Least Squares (GLS) to obtain efficient estimates. The GLS estimator is given by

$$\hat{\beta }={\left(X^{T}\Omega^{-1}X\right)}^{-1}X^{T}\Omega^{-1}P$$

We aim to test whether the effect of predictors ($D_{DRG}$ and $D_{Uterus}$) is decreasing and can be framed as a one-sided hypothesis test:

Null hypothesis: $H_{0}:\beta_{D}⩾0$; Alternative hypothesis: $H_{1}:\beta_{D}<0$

The test statistic for the coefficient is

$$t=\frac{{\hat{\beta }}_{D}}{SE\left({\hat{\beta }}_{D}\right)}$$

Under $H_{0}$, it follows a t-distribution with degrees of freedom approximated by n−p (where n is the number of observations and p is the number of estimated parameters). For a one-sided test, if the estimated coefficient ${\hat{\beta }}_{D}$ is negative, the p-value is computed as

$$p=P\left(T_{n-p}\leq t\right)$$

Where $T_{n-p}$ is subject to t-distribution with n−p degrees of freedom.

### Study design and statistics

The sample size in this study was not determined using statistical methods, but was guided by prior studies and literature in the field using similar experimental paradigms (36, 71). Mice with post-surgery complications, as defined by loss of >1g of body weight in 2 days after surgery, were euthanized before telemetry recording (n=2 total). One animal in *HoxB8 Piezo1/2* double knockout group was excluded from analysis due to an absence of labor signs on GD 20.

Data collection was conducted blindly, with post hoc registration to the respective condition for unbiased analysis. Animals with proprioceptive defects could still be identified and were therefore not truly blinded to the observer in this aspect. GraphPad Prism was used for statistical tests, unless specified otherwise. Sample sizes for each experiment are reported in the figure legends. All *in vivo* experiments were repeated at least twice or combined from at least two independent cohorts, yielding consistent results. All the horizontal lines in dot plots indicate median values unless otherwise specified.

## Supplementary Material

supplementary materials

Movie1

Supplementary Materials

Figs. S1 to S5

Movies S1

## Data Availability

The snRNA-seq data have been deposited at the Broad Institute Single Cell Portal under accession number SCP2924. Other data and scripts have been deposited on Zenodo (74, 84–86). All unique reagents generated in this study are available upon request to the corresponding author.
