# Supplementary material for: PIEZO channels link mechanical forces to uterine contractions in parturition
Source: Science. Author manuscript; Available in PMC 2026 Jan 15. (PMC12807505; doi:10.1126/science.ady3045)
Supplement: supplementary materials [file NIHMS2126701-supplement-supplementary_materials.pdf]

Science
AAAS

Supplementary Materials for

PIEZO channels link mechanical forces to uterine contractions in parturition

Yunxiao Zhang, Sejal A. Kini, Sassan A. Mishkanian, Oleg Yarishkin, Renhao Luo, Saba Heydari Seradj, Verina H. Leung, Yu Wang, M. Rocío Servín-Vences, William T. Keenan, Utku Sonmez, Manuel Sanchez-Alavez, Yuejia Liu, Xin Jin, Darren J. Lipomi, Li Ye, Michael Petrascheck, Antonina I. Frolova, Sarah K. England, Ardem Patapoutian

Corresponding author: ardem@scripps.edu

The PDF file includes:

Figs. S1 to S5

Other Supplementary Materials for this manuscript include the following:

Movies S1

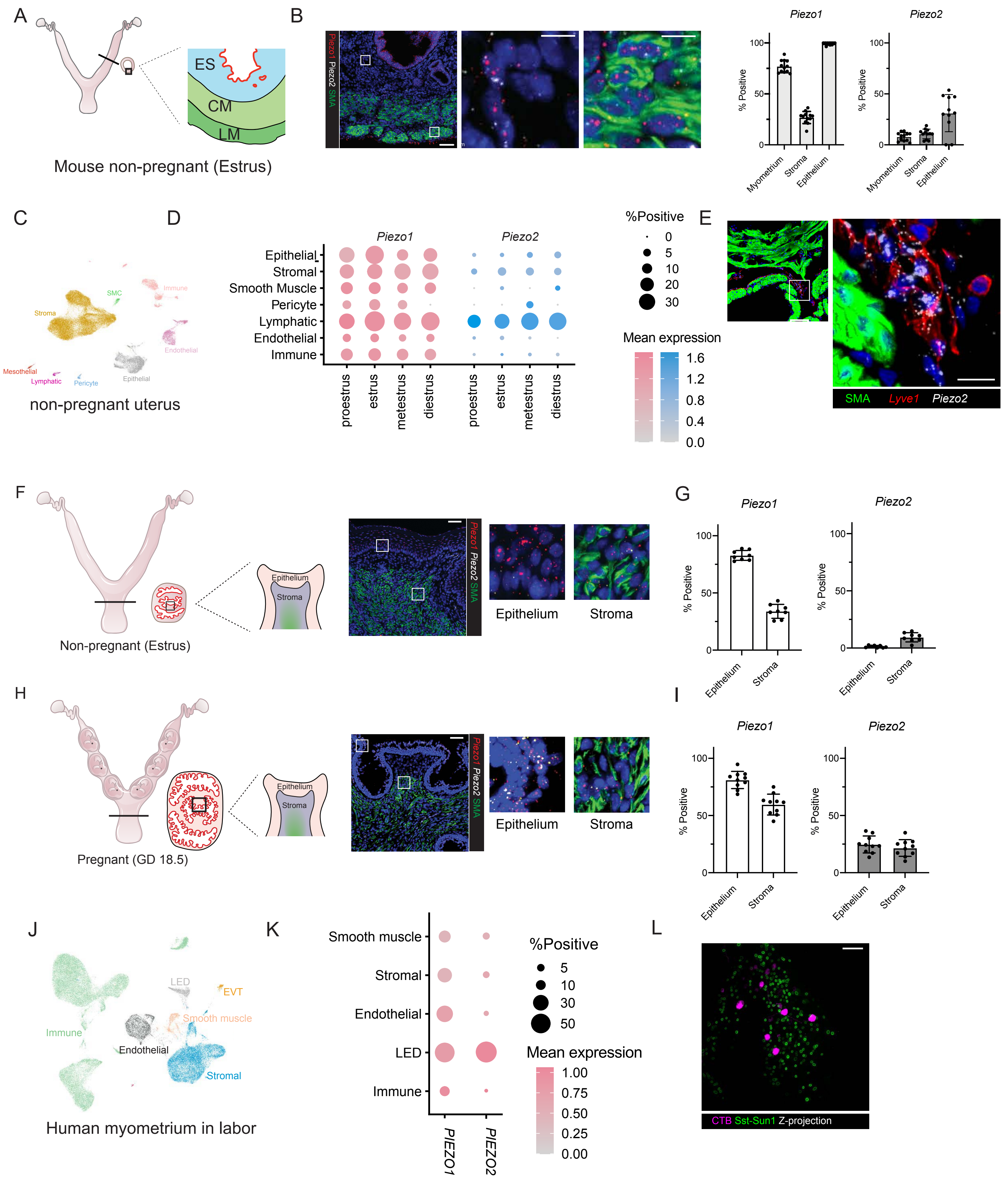
A
ES
CM
LM
Mouse non-pregnant (Estrus)
B
Piezo1 Piezo2 SMA
Piezo1
Piezo2
% Positive
Myometrium
Stroma
Epithelium
C
SMC
Immune
Stroma
Endothelial
Mesothelial
Lymphatic
Pericyte
Epithelial
non-pregnant uterus
D
Epithelial
Stromal
Smooth Muscle
Pericyte
Lymphatic
Endothelial
Immune
proestrus
estrus
metestrus
diestrus
%Positive
0
5
10
20
30
Mean expression
1.6
1.2
0.8
0.4
0.0
E
SMA Lyve1 Piezo2
F
Epithelium
Stroma
Non-pregnant (Estrus)
Piezo1 Piezo2 SMA
G
H
Pregnant (GD 18.5)
I
J
LED
EVT
Smooth muscle
Immune
Endothelial
Stromal
Human myometrium in labor
K
Smooth muscle
Stromal
Endothelial
LED
Immune
PIEZO1
PIEZO2
%Positive
5
10
30
50
Mean expression
1.00
0.75
0.50
0.25
0.00
L
CTB Sst-Sun1 Z-projection

Figure S1 *PIEZO1*/2 expression pattern in human and mouse reproductive system. (A) Schematic drawing of mouse uterus section taken from a non-pregnant animal at estrus stage. (B) *Piezo1/2* expression pattern in non-pregnant uterus is similar as the pregnant one. Scale bar: 50 µm in the overview, 10 µm in the inset. Images taken from 3 different animals. n=12 different images were quantified. (C) Published single cell sequencing dataset includes multiple cell types in the uterus. (D) *Piezo1* and *Piezo2* are expressed across multiple cell types and may have fluctuations according to the estrous cycle. (E) Lymphatic endothelial cells co-express *Piezo1* and *Piezo2* at high levels and are primarily localized between smooth muscle layers at GD 18.5. Scale bar: 50 µm. Images taken from 3 different animals. n=8 images were quantified. (F) Schematic drawing of mouse cervical section at estrus stage. (G) *Piezo1* is expressed in the non-pregnant cervix, while *Piezo2* is expressed at very low levels. Scale bar: 50 µm (H) Schematic drawing of mouse cervical section at late gestation. (I) In cervix at late pregnancy, *Piezo1* expression maintains while *Piezo2* expression is higher especially in the epithelium. Scale bar: 50 µm. Images taken from 3 different animals. n=10 images were quantified. (J) Single cell RNA-seq of human myometrium in labor reveals cell types in the myometrium. (K) *PIEZO1* is expressed in all major cell types, while *PIEZO2* is expressed almost only in lymphatic endothelial decidual cells (LEDs). (L) Retrograde labeling of uterus and cervix innervating DRG neurons with CTB revealed little overlap with *Sst*+ sensory neurons, the population that expresses *Piezo1*, suggesting that *Piezo1* expression may be rare in sensory innervation of the reproductive tract. Scale bar: 100 µm.

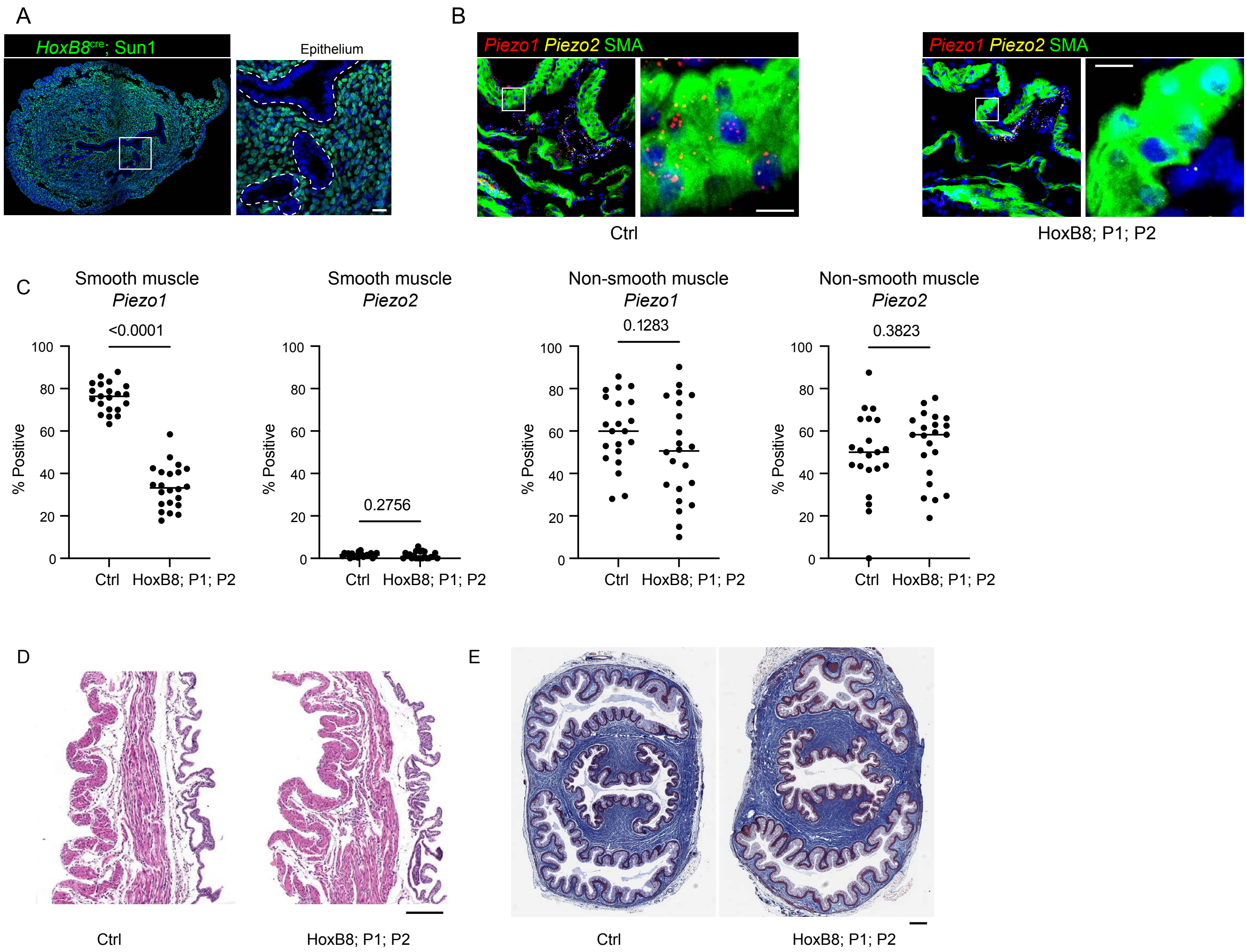

A
HoxB8cre; Sun1
Epithelium
B
Piezo1 Piezo2 SMA
Ctrl
Piezo1 Piezo2 SMA
HoxB8; P1; P2
C
Smooth muscle Piezo1
<0.0001
Smooth muscle Piezo2
0.2756
Non-smooth muscle Piezo1
0.1283
Non-smooth muscle Piezo2
0.3823
% Positive
0
20
40
60
80
100
Ctrl
HoxB8; P1; P2
D
Ctrl
HoxB8; P1; P2
E
Ctrl
HoxB8; P1; P2

Fig. S2. Histological characterization of *HoxB8*^{Cre} knockout. (A) *HoxB8*^{Cre} targets most cells in the non-pregnant uterus except the epithelium, as indicated by Rosa26 Sun1 reporter. Scale bar: 10 µm. (B) *HoxB8* ^{Cre} significantly reduced *Piezo1* expression in the uterine smooth muscle cells at gestational day 18.5 (Mann-Whitney test p-value<0.001), but non-smooth muscle cells that co-express *Piezo1* and *Piezo2* were unaffected (Mann-Whitney test p-value=0.1283 for *Piezo1*, 0.3823 for *Piezo2*), as quantified in (C). These double positive cells are mainly lymphatic endothelial cells. Scale bar: 50 µm, 10 µm in the inset. Images acquired from 3 animals for each genotype. n=21, 22 images for control and knockout groups, respectively. (D) The uterus at gestational day 18.5 had similar appearance between control and knockout animals with H&E staining. Scale bar: 200 µm. (E) Cervix sections at day 18.5 had similar appearance under trichrome staining between control and knockout animals. Scale bar: 200 µm.

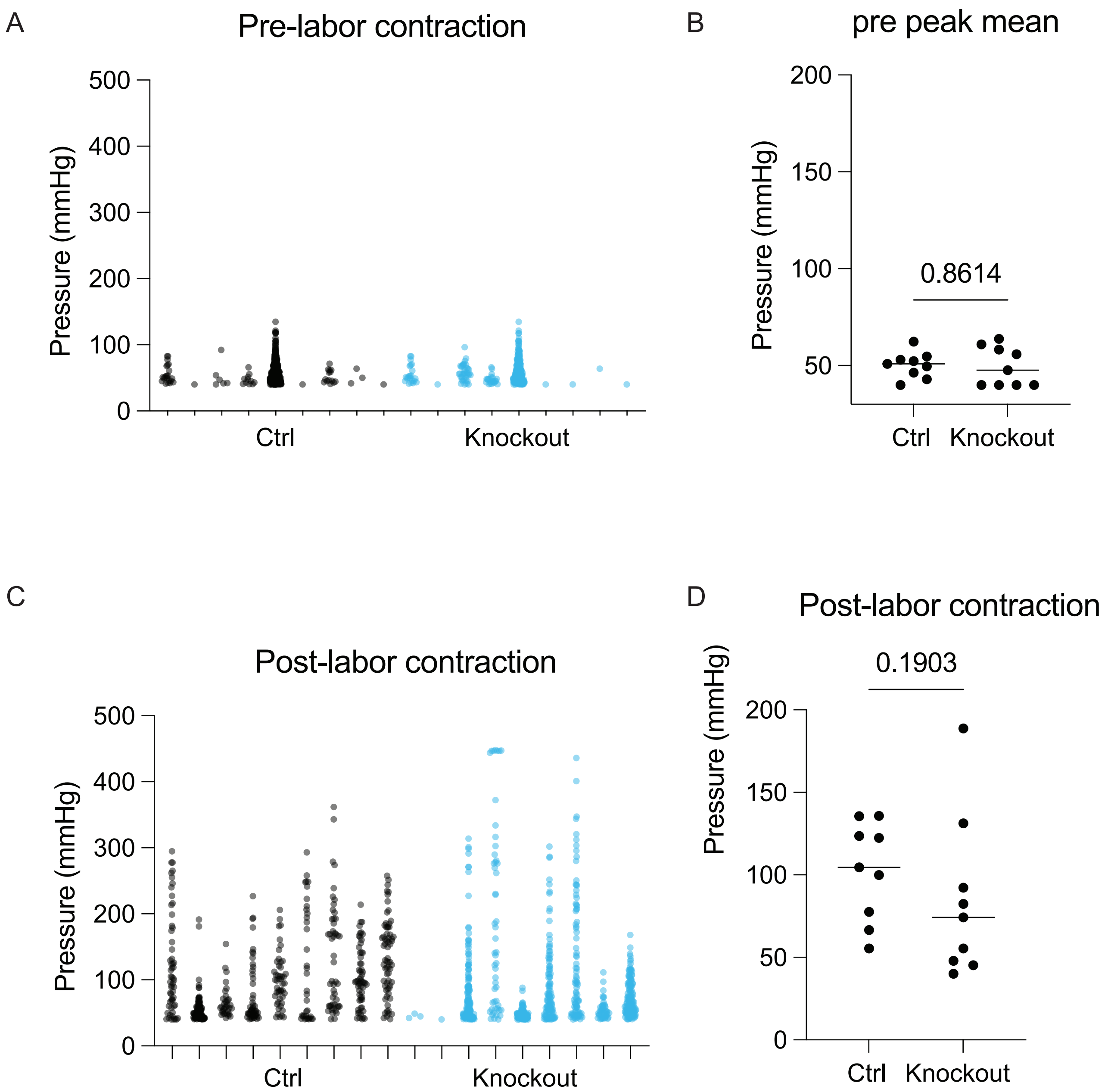
A
Pre-labor contraction
Pressure (mmHg)
500
400
300
200
100
0
Ctrl
Knockout
B
pre peak mean
Pressure (mmHg)
200
150
100
50
0.8614
Ctrl
Knockout
C
Post-labor contraction
Pressure (mmHg)
500
400
300
200
100
0
Ctrl
Knockout
D
Post-labor contraction
Pressure (mmHg)
200
150
100
50
0
0.1903
Ctrl
Knockout

Fig. S3. Contractions before and after labor in *HoxB8*^{Cre} knockout animals. (A) Pre-labor contractions (GD 18 midnight (ZT 18) to GD18 6 pm (ZT 12)) are similar between control and knockout animals as quantified in (B). (Mann-Whitney test p-value=0.8614). (C) Contractions 2-8 h after appearance of the first pup are similar between control and knockout animals, as quantified in (D). (Mann-Whitney test p-value=0.1903).

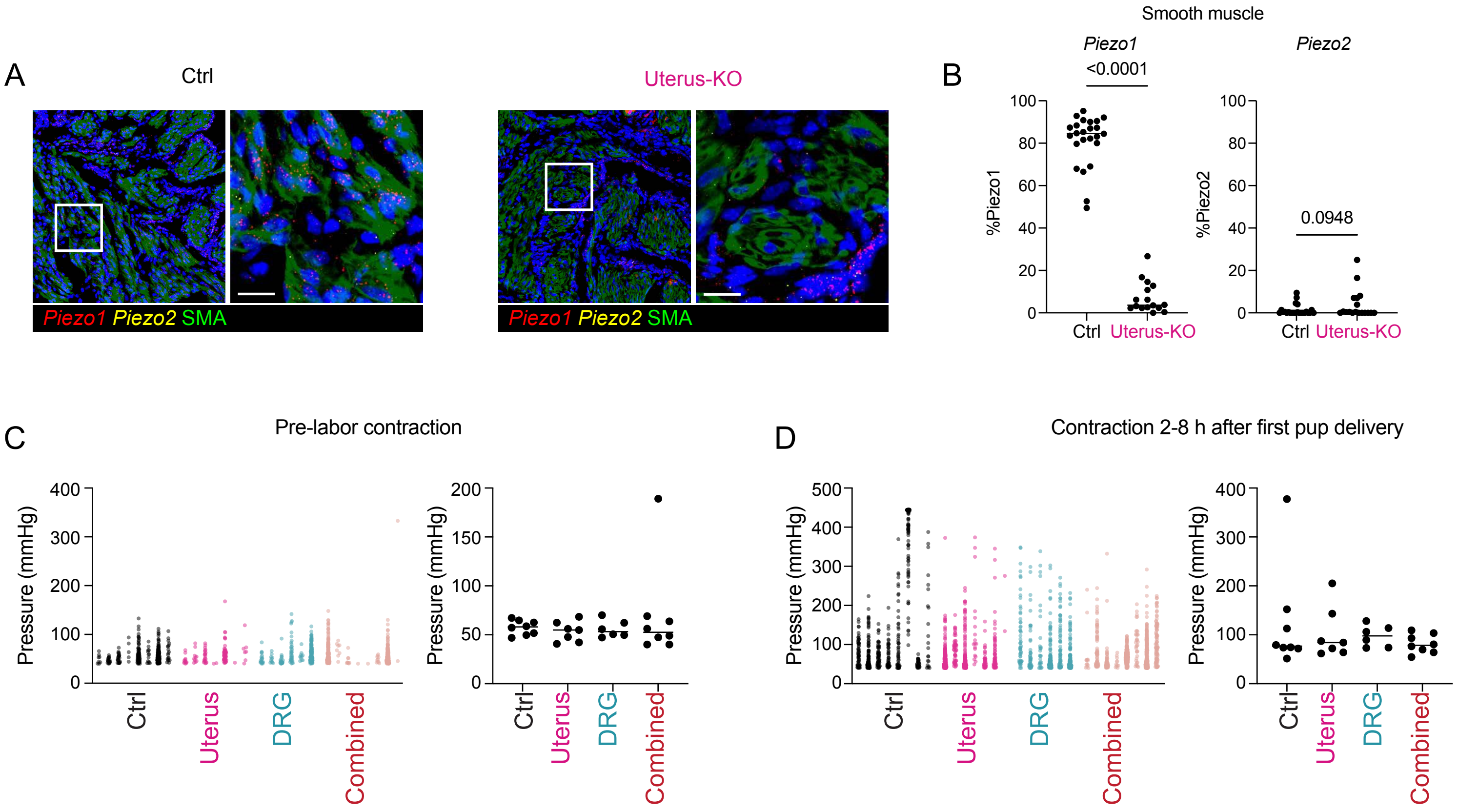

A
Ctrl
Uterus-KO
Piezo1 Piezo2 SMA
Piezo1 Piezo2 SMA
B
Smooth muscle
Piezo1
Piezo2
<0.0001
0.0948
%Piezo1
%Piezo2
Ctrl
Uterus-KO
Ctrl
Uterus-KO
C
Pre-labor contraction
Pressure (mmHg)
Pressure (mmHg)
Ctrl
Uterus
DRG
Combined
Ctrl
Uterus
DRG
Combined
D
Contraction 2-8 h after first pup delivery
Pressure (mmHg)
Pressure (mmHg)
Ctrl
Uterus
DRG
Combined
Ctrl
Uterus
DRG
Combined

Fig. S4. Contractions before and after labor in combined knockout animals. (A) Fluorescence ISH revealed that *Pgr* ^{cre} mediated effective deletion of *Piezo1* in uterine smooth muscle cells, as quantified in (B) (Mann-Whitney test p-value<0.0001). Scale bar: 20 µm. Images were taken for tissues from 3 different animals for each group. n=24, 16 images were quantified for control and knockout. (C) Pressure peaks from pre-labor contractions (GD 18 midnight (ZT 18) to GD18 6 pm (ZT 12)) were comparable across different treatment groups (Kruskal-Wallis test p-value=0.8430). (D) Pressure peaks at 2-8 hr after the first pup delivery were comparable across groups (Kruskal-Wallis test p-value=0.7239).

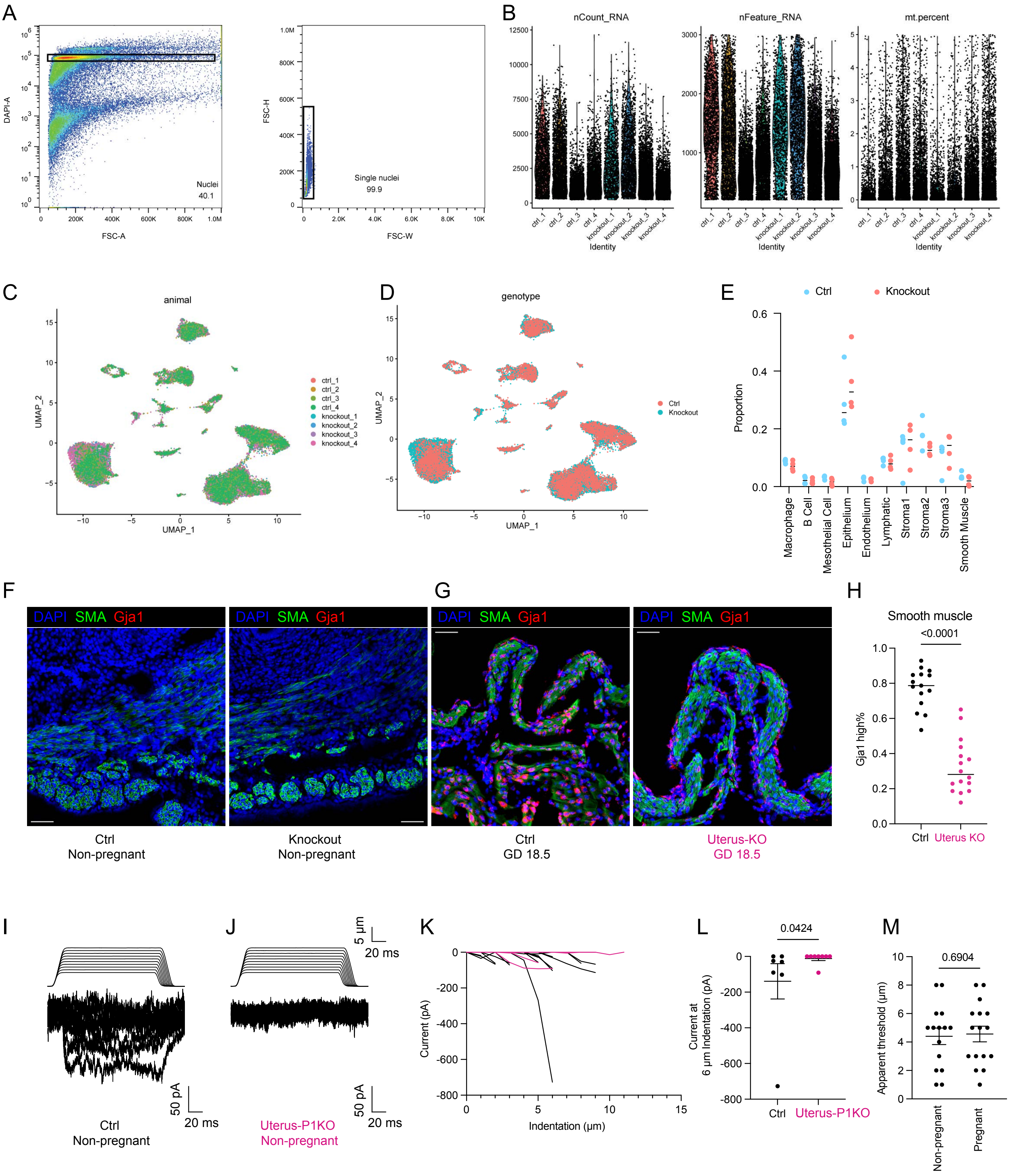
A
DAPI-A
FSC-A
Nuclei
40.1
FSC-H
FSC-W
Single nuclei
99.9
B
nCount_RNA
nFeature_RNA
mt.percent
Identity
ctrl_1
ctrl_2
ctrl_3
ctrl_4
knockout_1
knockout_2
knockout_3
knockout_4
C
animal
UMAP_1
UMAP_2
D
genotype
Ctrl
Knockout
E
Ctrl
Knockout
Proportion
Macrophage
B Cell
Mesothelial Cell
Epithelium
Endothelium
Lymphatic
Stroma1
Stroma2
Stroma3
Smooth Muscle
F
DAPI SMA Gja1
Ctrl
Non-pregnant
Knockout
Non-pregnant
G
Ctrl
GD 18.5
Uterus-KO
GD 18.5
H
Smooth muscle
<0.0001
Gja1 high%
Ctrl
Uterus KO
I
50 pA
20 ms
Ctrl
Non-pregnant
J
5 µm
20 ms
50 pA
20 ms
Uterus-P1KO
Non-pregnant
K
Current (pA)
Indentation (µm)
L
0.0424
Current at 6 µm Indentation (pA)
Ctrl
Uterus-P1KO
M
0.6904
Apparent threshold (µm)
Non-pregnant
Pregnant

Figure S5. Single nuclei sequencing revealed difference in *Gja1* expression. (A) The samples were gated based on DAPI in FACS to obtain nuclei and further filtered based on forward scattering light to obtain single nuclei. (B) Read depth, and sample quality metrics of the single nuclei sequencing samples are shown as violin plots. (C) Cells from different animals or different batches overlapped in UMAP representation without obvious batch effect. (D) The cells from control or knockout animals intermingled in the clusters, suggesting that knockout of *Piezo1/2* does not change major tissue composition, which is further confirmed by the proportions of the major cell types in (E). (F) Non-pregnant uterus from control or knockout animals had no detectable levels of *Gja1*. Scale bar: 50 µm. (G) *Gja1* expression in uterus-specific knockout was reduced at GD 18.5, as quantified in (H) (Mann-Whitney test p-value<0.0001). Images were quantified from sections belonging to 3 different animals for each genotype. n=15, 16 sections for control and knockout group, respectively. Representative traces of mechanically activated current recorded from cultured smooth muscle cells from control (I) and uterus-specific knockout animals (J). (K) Mechanical response was diminished in smooth muscle cells from knockout animals, as quantified in (L) (Mann-Whitney test p-value=0.0424). Smooth muscle cells were isolated from 2 animals for each genotype. n=15, 11 cells for control and knockout, respectively. (M) Apparent threshold for indentation-invoked current is similar between pregnant and non-pregnant conditions in cells from control animals (Mann-Whitney test p-value=0.6904).

Movie S1.
Light sheet imaging of *Piezo2*+ innervation in the mouse reproductive tract.
